# The Sewing-Machine and Health

**Published:** 1904-12

**Authors:** 


### THE SEWING-MACHINE AND HEALTH.

Nussbaum (*Wiener klinische Rundsschan*) refers to statistics and his own experience to show that the position at the machine and the constant use of the legs are liable to cause congestion in the female genitalia and consequent inflammation. The menses are frequently very irregular, and abortions, inflammation of the adnexa and other gynecologic troubles are unusually prevalent in this class of working-women. He advises that they should take especial pains to sit erect and breathe deep, frequently rising to change their occupation or to step out of doors or practice respiratory gymnastics. Good ventilation and illumination are particularly necessary for persons who have to use the sewing-machine constantly.—*Medical Review of Reviews.*

